# Deep learning-based automatic segmentation and CT elastography radiomics for preoperative prediction of right recurrent laryngeal nerve lymph node metastasis in esophageal cancer

**DOI:** 10.3389/fonc.2026.1748391

**Published:** 2026-04-24

**Authors:** Chao Ji, Qingqing Li, Shumin Jiang, Lingling Wang, Sunkui Ke, Feng Wang, Hongbin Duan, Xiaomei Lin, Xi’e Xu, Xiaoli Huang

**Affiliations:** 1 Department of Thoracic Surgery, Zhongshan Hospital Xiamen University, School of Medicine, Xiamen University, Xiamen, China; 2 Xiamen University, Xiamen, China; 3 Department of Cardiology Division1, Xiamen Cardiovascular Hospital, Xiamen University, Xiamen, China; 4 Fujian Provincial Cancer Hospital, Fuzhou, China; 5 Department of Gastroenterology, Zhongshan Hospital Xiamen University, School of Medicine, Xiamen University, Xiamen, China; 6 Department of Vascular Surgery, Zhongshan Hospital, Xiamen University School of Medicine, Xiamen University, Xiamen, China

**Keywords:** computed tomography elastography image, esophageal cancer, nnU-Net, radiomics, recurrent laryngeal nerve

## Abstract

**Objective:**

Accurate preoperative prediction of lymph node metastasis adjacent to the right recurrent laryngeal nerve (RLN) in esophageal squamous cell carcinoma (ESCC) is crucial for treatment planning. This study aimed to develop and validate an imaging approach that integrates deep learning-based automatic segmentation (nnU-Net) with computed tomography (CT)-derived differential elasticity map (DEM) to predict RLN lymph node metastasis in ESCC.

**Methods:**

This retrospective study included 415 patients diagnosed with ESCC. An automatic segmentation model was trained using the nnU-Net framework to delineate lymph nodes near the right RLN. Three-dimensional CT elasticity images were generated from segmented CT voxels, from which radiomic features, including first-order entropy and multi-scale fractal dimensions, were extracted. Statistically significant features were selected using statistical tests and area under the curve (AUC) analyses, and their diagnostic efficacy, probability calibration, and clinical decision-making value were further evaluated in a validation cohort.

**Results:**

The automatic segmentation model achieved a Dice coefficient of 0.898 ± 0.024. Five DEM-derived radiomic features were ultimately selected: one first-order entropy feature (E_original_firstorder_Entropy) and four fractal dimension-related features. The entropy feature exhibited the highest diagnostic performance (AUC = 0.814, sensitivity = 0.895, specificity = 0.709), and fractal dimension-related features were significantly elevated (all P < 0.001) in the metastatic group, indicating increased textural complexity and multi-scale irregularity. Calibration curves demonstrated the robustness of entropy-based probability estimation. Decision curve analysis confirmed the clinical utility of these features, showing positive net benefits across a wide range of threshold probabilities (10%–70%).

**Conclusion:**

The proposed automated workflow, combining nnU-Net segmentation and DEM-based radiomics, enables accurate and non-invasive prediction of right RLN lymph node metastasis in ESCC. First-order entropy and fractal dimension features offer valuable complementary information beyond conventional radiomics, providing a promising tool for preoperative decision support and personalized treatment planning.

## 1 Background

Esophageal cancer (EC) is a common gastrointestinal malignancy, with an incidence rate of 2.06% ranking the 11th among all cancers worldwide and a cancer-related mortality rate of 4.6% ranking 7th (1). China is a high-incidence region for EC, accounting for over 50% of global cases (2). Due to insidious early-stage symptoms, most patients are diagnosed at a locally advanced stage or with distant metastasis. Among metastatic sites, lymph nodes adjacent to the right recurrent laryngeal nerve (RLN) are the most common for thoracic esophageal squamous cell carcinoma (ESCC) (3). Lymph node metastasis status directly affects patient prognosis and the selection of treatment strategies—patients without lymph node metastasis have a significantly higher 5-year survival rate than those with metastasis (4, 5). Therefore, this study aims to achieve accurate preoperative assessment of right RLN lymph node metastasis in EC to formulate personalized treatment strategies.

Currently, the clinical diagnosis of right RLN lymph node metastasis relies primarily on CT. However, conventional CT judges solely based on basic features such as lymph node size (short-axis diameter > 1 cm) and morphology, which easily leads to missed diagnosis of micrometastases with a short-axis diameter < 5 mm (6). Thus, its sensitivity and specificity are insufficient to meet clinical needs. In recent years, with advances in imaging technology, CT radiomics has emerged as an analytical method that provides new insights for predicting lymph node metastasis (7, 8). It extracts high-dimensional quantitative features (e.g., morphology, density, texture) from CT images via automated algorithms. Previous studies have confirmed that CT radiomic features of EC-associated lymph nodes can effectively distinguish between metastatic and non-metastatic nodes, achieving a higher area under the receiver operating characteristic curve (AUC) compared to conventional CT methods (9, 10).

Nevertheless, existing research has two key limitations: first, radiomics is highly dependent on manual or semi-automatic delineation of the region of interest (ROI), which is time-consuming, labor-intensive, subject to significant operator bias, and lacks reproducibility (11), making it difficult to translate into routine clinical practice; second, traditional radiomic features only focus on the intensity and texture patterns of CT voxels, ignoring changes in tissue biomechanical properties (e.g., elastic heterogeneity) caused by tumor infiltration—these properties are important indicators reflecting the pathological state of tissues (12). Therefore, this study focuses on such elastic features (e.g., entropy, fractal dimension) to determine the presence of tumor metastasis by describing and analyzing differences in tissue characteristics.

Notably, the aforementioned limitations are prevalent in clinical oncology, such as in the diagnosis of EC. These methodological drawbacks are driving the advancement and integration of tumor imaging technologies and computational methods. For example, Weng et al. (13) combined YOLO models with hyperspectral imaging to optimize the detection efficiency of EC lesions; Chang et al. (14) improved the detection rate of early EC using spectral imaging technology; Chen et al. (15) optimized the band selection strategy for the spectrum-aided visual enhancer (SAVE), providing a new scheme for tumor visualization. These studies collectively confirm that combining advanced imaging technologies with automated computational methods is an effective approach to overcoming current clinical research limitations, which also provides a meaningful methodological foundation for our study.

Building on the aforementioned clinical needs, research gaps, and methodological advancements in advanced imaging technologies and automated computing, this study aims to predict right RLN lymph node metastasis in EC by combining CT elastography (CTE) with an automatic segmentation model. We utilize the nnU-Net architecture to develop a fully automatic lymph node segmentation model, addressing the poor reproducibility of traditional manual segmentation. Meanwhile, based on segmented CT voxels, we generate Differential Elasticity Maps (DEMs) through mathematical modeling and geometric analysis, extracting first-order elastic features with affine-invariant properties. These features are unaffected by parameters such as scan slice thickness and reconstruction algorithms, enabling stable reflection of the elastic heterogeneity of local soft tissues (12). Finally, we integrate clinical data and elastic features to construct a prediction model, achieving non-invasive and accurate assessment of right RLN lymph node metastasis status. This study provides a more reliable reference for accurate preoperative staging and treatment planning of EC.

## 2 Methods

### 2.1 Patients data

**Figure 1:**
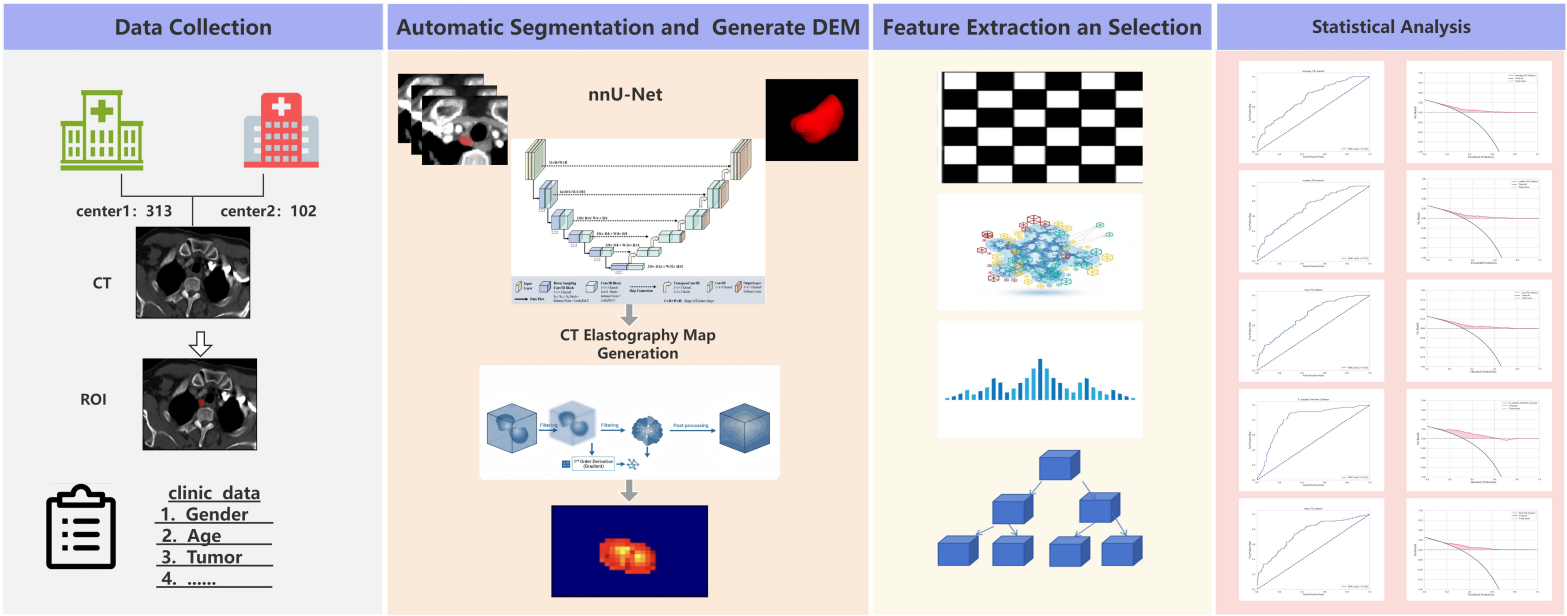
Four-step workflow diagram for medical image analysis. Step one,data collection, shows two hospitals, CT scans, and clinical data. Step two, automatic segmentation, illustrates nnU-Net architecture, elastography map generation, and tumor segmentation. Step three, feature extraction, displays patterns, cluster plots, histogram,and a decision tree. Step four, statistical analysis, consists of several line and curve graphs for data comparison.

The Ethics Committee of Zhongshan Hospital Affiliated to Xiamen University approved the experiment. All methods were carried out in accordance with all relevant guidelines and regulations. Informed consent was obtained from all participants and/or their legal guardians. The methodology of this investigation is depicted in **Figure 1**. This study retrospectively enrolled 415 patients with ESCC from two medical centers in China. The training cohort comprised 313 patients treated at Zhongshan Hospital affiliated with Xiamen University from May 2015 to May 2025. The external validation cohort included 102 patients treated at Fujian Cancer Hospital between May 2018 and May 2025. Inclusion criteria: (1) Undergoing standard radical esophagectomy and corresponding lymphadenectomy; (2) Pathologically confirmed ESCC; (3) Completing contrast-enhanced chest CT within one week before surgery; (4) Surgery as initial treatment without neoadjuvant chemoradiotherapy. Exclusion criteria: (1) Preoperative neoadjuvant therapy; (2) Concurrent malignancies; (3) Incomplete imaging data or critical clinical information. A total of 415 eligible patients were enrolled, including 313 in the training cohort and 102 in the external validation cohort, with pathological confirmation of right RLN lymph node metastasis as the gold standard for labeling, ensuring the reliability of the research grouping.

### 2.2 Automatic segmentation model training

The lymphatic node segmentation task was trained using the nnU-Net architecture. Autoconfigured preprocessing steps included automatic cropping of image borders, resampling to the same resolution as the gold standard segmentation masks, and Z-score normalization. Training was performed using “2D,” “3D full resolution,” and “3D full resolution cascade” configurations. For each training process, the training dataset was randomly divided into five folds for cross-validation, resulting in five different models. Each fold was trained for 1000 epochs. The predictions from the five models were integrated to obtain the final output. The best-performing model, based on the highest average Dice Similarity Coefficient (DSC) value on the validation set, was selected.

### 2.3 CT elastography image generation

We generated a CT-based Differential Elasticity Map (DEM) that is invariant to local affine deformations and reflects micro-local tissue elasticity, based on the methodology described by W. Cao et al. (12)(doi: 10.1007/s10278-024-01178-8). The core assumption underlying the construction of CT-based Differential Elasticity Map (DEM) is that soft tissue undergoes approximately local affine deformation, which is the theoretical basis for calculating the affine-invariant scalar elasticity response E = (▽I)H^-^¹(▽I)T (Cao et al., 2025), and ensures that the extracted elastic features reflect the intrinsic biomechanical properties of lymph node tissue rather than geometric artifacts.

Another key assumption is that the light Gaussian smoothing applied in preprocessing only suppresses image noise without distorting the edge and microstructural information of lymph nodes. In addition, we assume that the consistent standardized preprocessing and feature extraction pipeline applied to all cases can eliminate individual differences in imaging data and ensure the comparability of radiomic features. The process involves the following key steps:

#### 2.3.1 ROI preparation

The 3D lymph node ROI, segmented by nnU-Net, was extracted. The CT volume was resampled to isotropic voxels (1x1x1 mm^3^) using trilinear interpolation.

#### 2.3.2 Preprocessing

Light Gaussian smoothing (σ = 1 voxel) was applied to reduce noise while preserving edges.

#### 2.3.3 Derivative computation

First- and second-order spatial derivatives were computed using Gaussian-derivative filters to obtain the gradient vector (▽I) and the Hessian matrix (H) for each voxel.

#### 2.3.4 Affine-invariant elasticity response

Under the assumption that soft tissue deforms locally in an approximately affine manner, a scalar elasticity response (E) was computed by combining the gradient and Hessian information: E = trace((H + ϵ I)^(-1) * (▽I ▽I^T)), where a small regularization term ϵ is added to the Hessian to ensure numerical stability in regions with poorly conditioned second-order structure.

#### 2.3.5 Post-processing

The resulting response map E typically has a long-tailed distribution. A monotonic root-based compression (e.g., square root) was applied to enhance subtle structural variations and suppress extreme outliers. Finally, linear normalization was performed to scale values to a fixed range [0, 255].

The final output is a 3D DEM aligned with the original CT, emphasizing tissue mechanical behavior rather than raw intensity. The DEM generation method described above has been integrated into the PixelMed AI platform, facilitating reproducible research and clinical translation.

### 2.4 Feature extraction and selection

The open-source Pyradiomics package (http://pypi.org/project/pyradiomics/) based on Python 3.8 was used to extract first-order features. The fractal dimension feature extraction steps were as follows: First, all non-zero voxels were extracted from the image to remove background noise and focus on the target region. Then, a logarithmic scale vector using powers of 2 was constructed for multi-scale analysis, with the scale range determined by the maximum size of the image. Next, the three-dimensional box-counting algorithm was applied: boxes with pseudo-random offsets were placed in the image space, and the number of voxels within each box was counted. This process was repeated multiple times to reduce offset-induced randomness, and the results were averaged per scale. A linear regression was then fitted to the relationship between voxel count and scale in log-log coordinates to identify the optimal fitting window (“fractal scale window”), considering the adjusted R² coefficient. The fractal dimension was calculated as the negative slope of the regression line. Subsequently, statistical analysis was performed on the fractal dimension, including mean, median, maximum, and minimum, to comprehensively characterize the image’s fractal properties.

In addition to these radiomic features, we also calculated curvature-based topological features. Curvature properties describe the local geometric shape of a surface, including bending degree and direction. Gaussian curvature distinguishes concave, convex, and flat regions; mean curvature measures local bending trend; principal curvatures capture extreme bending in specific directions. Higher-level metrics such as curvature intensity, sharpness, shape index, and total curvature were derived to support shape analysis and feature extraction.

To select features with good reproducibility and low redundancy, features with P > 0.05 were removed, and then features with an AUC > 0.7 were retained.

### 2.5 Statistical analysis

All statistical analyses were performed using Python (version 3.8.2; https://www.python.org). Independent samples t-tests were used to analyze normally distributed quantitative data, with continuous variables expressed as mean ± standard deviation. The Mann-Whitney U test was used to analyze non-normally distributed data, expressed as median (interquartile range). Levene’s test was used to assess the homogeneity of variance. Comparisons of categorical data were performed using the chi-square test or Fisher’s exact test, and categorical variables were represented as counts (n) and percentages (%). Statistical significance was set at P < 0.05. The classification performance of features was evaluated using ROC curves and the area under the curve (AUC). Decision curve analysis (DCA) was performed to assess the clinical value of the model by quantifying the net benefit at different threshold probabilities. Differences between AUCs of different features were assessed using the DeLong test. Calibration of the feature model was assessed using calibration curves, analyzing the consistency between the feature’s predicted probabilities and the actual incidence. The degree of feature calibration was quantified using the Hosmer-Lemeshow (HL) test, with P > 0.05 indicating good model fit.

## 3 Results

### 3.1 Patient clinical outcomes

A total of 415 patients were enrolled in this study, with an overall age of 61.9 ± 8.2 years. Among them, there were 330 males (79.5%) and 85 females (20.5%), including 282 cases in the negative group and 133 cases in the positive group. There was no statistically significant difference in age between the two groups (P = 0.719), and the difference in gender distribution was also not significant (P = 0.267). However, the distribution of smoking status differed between the two groups (P<0.001), while the difference in Tumor location distribution was not significant (P = 0.282).

The Tumor longest diameter was significantly larger in the positive group (median 4.0 [3.5, 4.9] vs. 3.5 [2.6, 4.8], P<0.001), and there was also a significant difference in the degree of differentiation between the two groups (P<0.001). For CT-based differential elasticity indices, LN Longest diameter was significantly increased in the positive group (1.2 [0.9, 1.5] vs. 0.6 [0.5, 0.7], P<0.001). Regarding CT quantitative indicators, CT value of LN plain scan was slightly higher in the positive group (P = 0.033), while Enhanced CT value of LNs and Enhanced CT value of tumor were lower in the positive group (both P<0.001), and CT value of tumor plain scan was increased in the positive group (P<0.001). These findings suggest that positive lesions exhibit distinct imaging features in terms of intensity distribution and texture configuration.

### 3.2 Automatic segmentation and select DEM features

**Figure 2:**
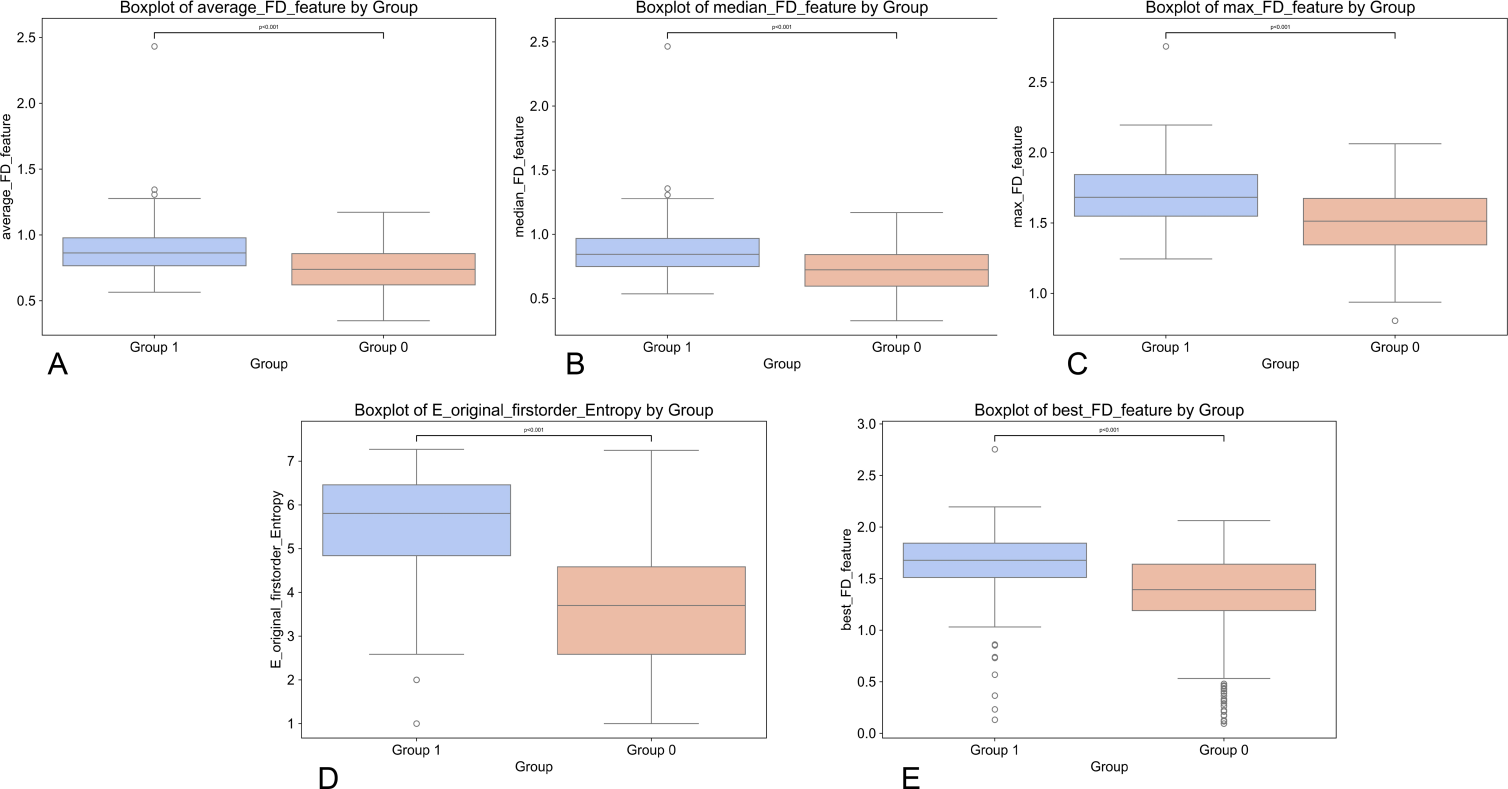
Five boxplots labeled **(A–E)** compare statistical features between Group 1 and Group 0. Each plot displays distribution differences in features: average_FD,median_FD, max_FD, entropy, and best_FD, with all p-values less than 0.001 indicating significant group differences.

The above overall and subgroup baseline statistics are presented in **Table 1** and **Figure 2**. The Dice Similarity Coefficient (DSC) for lymph node segmentation in the test set was 0.898 ± 0.024. In Differential Elasticity Map (DEM)-driven radiomics analysis, feature selection was initially performed based on a P-value threshold (P > 0.05 excluded) and a discrimination criterion (AUC > 0.7), ultimately retaining five robust features with low redundancy (**Table 2**). E_original_firstorder_Entropy was significantly higher in the positive group than in the negative group (5.8 [4.8, 6.5] vs 3.7 [2.6, 4.6], P < 0.001), and four fractal dimension-related features (max_FD_feature, best_FD_feature, average_FD_feature, median_FD_feature) were also consistently elevated in the positive group (all P < 0.001). This result indicates that, compared to negative lesions, positive lesions exhibit higher textural complexity and multiscale irregularity in the DEM space, manifested as a systematic increase in entropy and fractal dimension, thereby supporting the existence of “elasticity-morphology” coupling differences.

**Table 1:** Baseline feature statistics.

Feature		Overall(415)	Label=0(282)	Label=1(133)	P-Value
Gender, n (%)	Male	330 (79.5)	229 (81.2)	101 (75.9)	0.267
	Female	85 (20.5)	53 (18.8)	32 (24.1)	
Age (years) mean (SD)		61.9 (8.2)	61.8 (7.9)	62.1 (8.9)	0.719
Smoking history, n (%)	No	283 (68.2)	196 (69.5)	87 (65.4)	<0.001
	Yes	132(31.8)	86 (30.5)	46 (34.6)	
Tumor Longest diameter (cm) median [Q1,Q3]		3.7[2.8,4.9]	3.5 [2.6,4.8]	4.0 [3.5,4.9]	<0.001
Tumor location, n (%)	Upper	22 (5.3)	12 (4.3)	10 (7.5)	0.282
	Middle	277 (66.7)	187 (66.3)	90 (67.7)	
	Lower	116 (28.0)	83 (29.4)	33 (24.8)	
Degree of differentiation[Q1,Q3]	Low	24 (5.8)	7 (2.5)	17 (12.8)	<0.001
	Medium	371 (89.4)	262 (92.9)	109 (82.0)	
	High	20 (4.8)	13 (4.6)	7 (5.3)	
LN Longest diameter (cm), median [Q1,Q3]		0.7 [0.5,1.1]	0.6 [0.5,0.7]	1.2 [0.9,1.5]	<0.001
CT value of LN plain scan, mean (SD)		35.0 (21.8)	33.6 (23.3)	38.0 (17.8)	0.033
Enhanced CT value of LNs, mean (SD)		59.8 (17.1)	62.2 (15.5)	54.7 (19.3)	<0.001
CT value of tumor plain scan, median [Q1,Q3]		22.1 [9.2,36.2]	17.8 [5.6,33.6]	27.8 [20.1,37.1]	<0.001
Enhanced CT value of tumor, mean (SD)		38.1 (10.5)	39.6 (10.8)	34.9 (9.3)	<0.001

**Table 2:** Statistics of filtered radiomics features.

Feature	Overall(415)	Label=0(282)	Label=1(133)	P-Value
E_original_firstorder_Entropy, median [Q1,Q3]	4.2 [3.0,5.8]	3.7 [2.6,4.6]	5.8 [4.8,6.5]	<0.001
max_FD_feature, median [Q1,Q3]	1.6 [1.4,1.7]	1.5 [1.3,1.7]	1.7 [1.5,1.8]	<0.001
best_FD_feature, median [Q1,Q3]	1.5 [1.3,1.7]	1.4 [1.2,1.6]	1.7 [1.5,1.8]	<0.001
average_FD_feature, median [Q1,Q3]	0.8 [0.7,0.9]	0.7 [0.6,0.9]	0.9 [0.8,1.0]	<0.001
median_FD_feature, median [Q1,Q3]	0.8 [0.6,0.9]	0.7 [0.6,0.8]	0.8 [0.7,1.0]	<0.001

### 3.3 ROC analysis of single features

**Figure 3:**
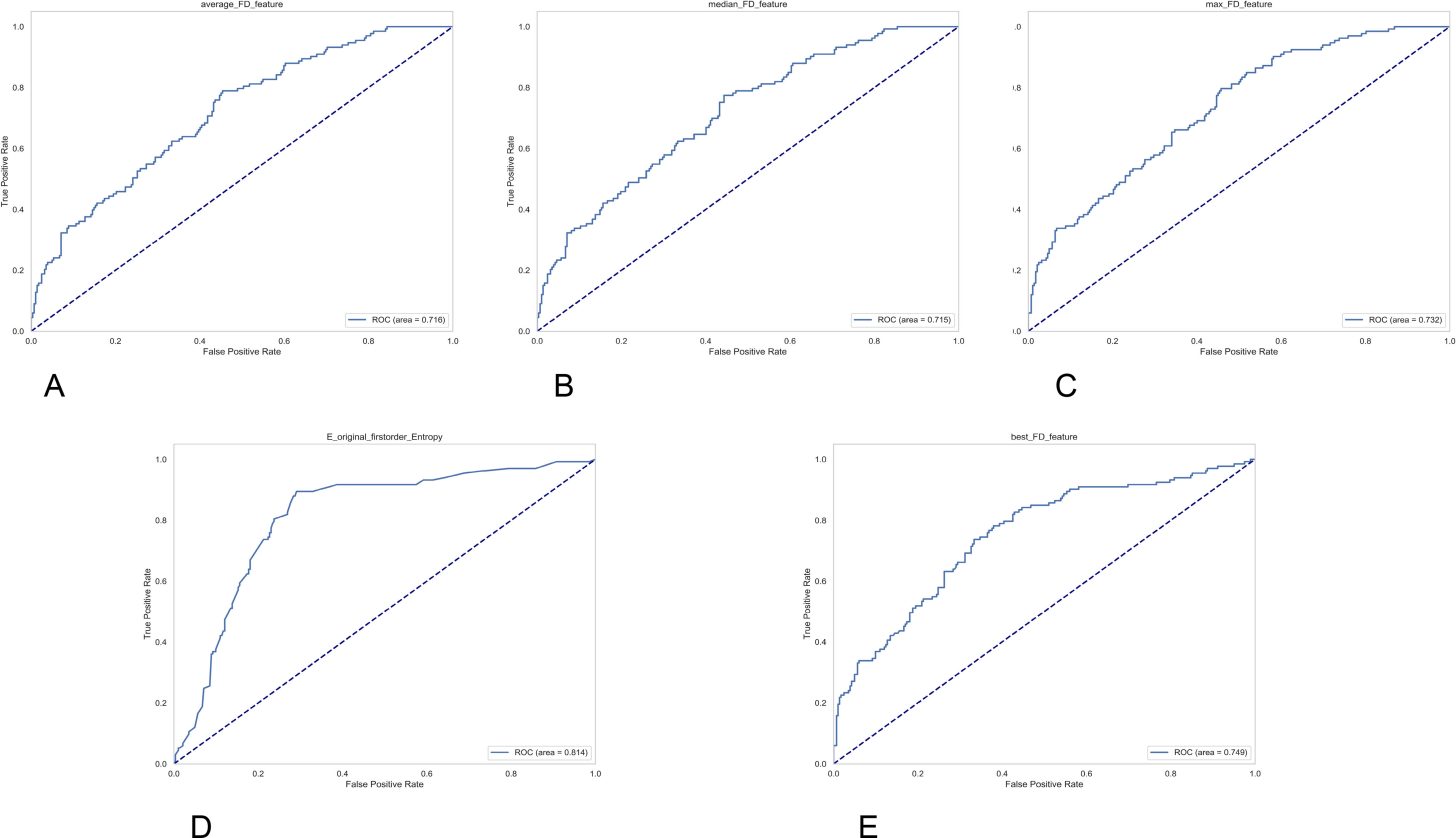
Five ROC curve graphics labeled (A-E), each displaying true positive rate versus false positive rate for different features. **(A)** shows average_FD_feature (AUC 0.716), **(B)** shows median_FD_feature (AUC 0.715), **(C)** shows max_FD_feature (AUC 0.732), **(D)** shows E_original_firstorder_Entropy (AUC 0.814), and **(E)** shows best_FD_feature (AUC 0.749). Each plot includes a blue ROC curve above the diagonal reference line.

In terms of diagnostic efficacy, ROC analysis of single features showed that entropy features had the best overall discriminative performance, with an AUC of 0.814 (**Figure 3**). At a threshold of 4.392, it achieved a sensitivity of 0.895, a specificity of 0.709, an accuracy of 0.769, and an F1-score of 0.713 (see **Table 3**). Fractal dimension features generally exhibited moderate discriminative power; the AUC for best_FD_feature was 0.749, max_FD_feature was 0.732, and average_FD_feature and median_FD_feature were 0.716 and 0.715, respectively. These features generally showed a profile of high sensitivity but relatively low specificity, suggesting that they are more sensitive to identifying positive cases but may be accompanied by a certain false-positive rate.

**Table 3:** Diagnostic efficacy of screened radiomic features.

Feature	Threshold	ACC	AUC	Sensitivity	Specificity	NPV	PPV	F1
E_original_firstorder_Entropy	4.392	0.769	0.814	0.895	0.709	0.935	0.592	0.713
best_FD_feature	1.535	0.689	0.749	0.737	0.667	0.843	0.51	0.603
max_FD_feature	1.535	0.624	0.732	0.797	0.543	0.85	0.451	0.576
average_FD_feature	0.751	0.624	0.716	0.789	0.546	0.846	0.451	0.574
median_FD_feature	0.736	0.627	0.715	0.774	0.557	0.84	0.452	0.571

### 3.4 Feature probability calibration performance

**Figure 4:**
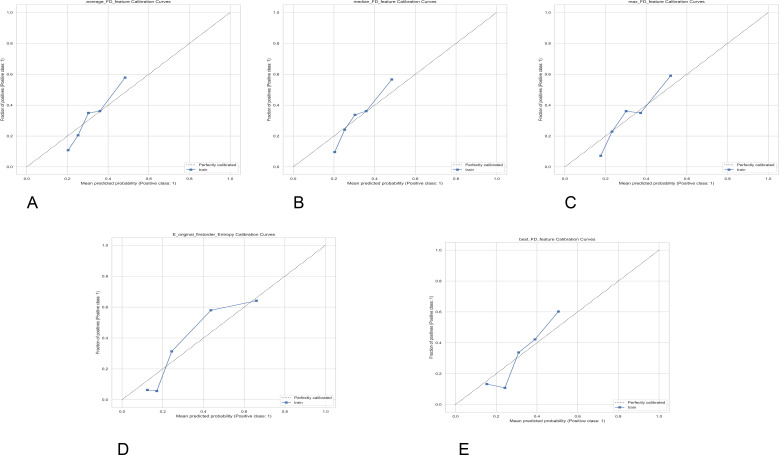
Five-panel grid of calibration curve line charts labeled **(A–E)**, each comparing mean predicted probability to observed fraction of positives for different prediction models. All panels include a diagonal reference line labeled “Perfectly calibrated” and a blue line showing model performance on training data. Axes are consistently labeled “Mean predicted probability (Positive class: 1)” on the x-axis and “Fraction of positives (Positive class: 1)” on the y-axis. Each chart displays minor deviations of model lines from the perfect calibration line. Panel titles are: average_FD_feature, median_FD_feature, max_FD_feature, E_original_firstorder_Entropy, and best_FD_feature Calibration Curves.

Regarding single-feature probability calibration, the calibration curves for each feature generally exhibit a monotonically increasing trend from low to high predicted probabilities, suggesting that the output probabilities possess basic interpretability (**Figure 4**). The fractal dimension-related features show good agreement with the ideal line in the moderate probability range, with max_FD_feature having the smallest deviation in the interval of approximately P = 0.3-0.6. In contrast, average_FD_feature and median_FD_feature exhibit a slight under-prediction at the low probability end p<0.25 and a slight over-prediction trend after entering the medium-high probability end P = 0.4-0.6. Comparatively, E_original_firstorder_Entropy is generally distributed along the diagonal line in the low-to-medium-high probability range, demonstrating the best consistency and stability. best_FD_feature is slightly below the ideal line at the low probability end, gradually approaching the diagonal line as the probability increases, and achieves good fitting in the medium-high probability range. Combined with the Hosmer–Lemeshow test results (if P>0.05), the above patterns indicate that the entropy feature’s probability estimation is the most robust, while the fractal dimension features exhibit small, consistent-direction systematic biases in different probability segments, but all within an acceptable range.

### 3.5 DCA of single features

**Figure 5:**
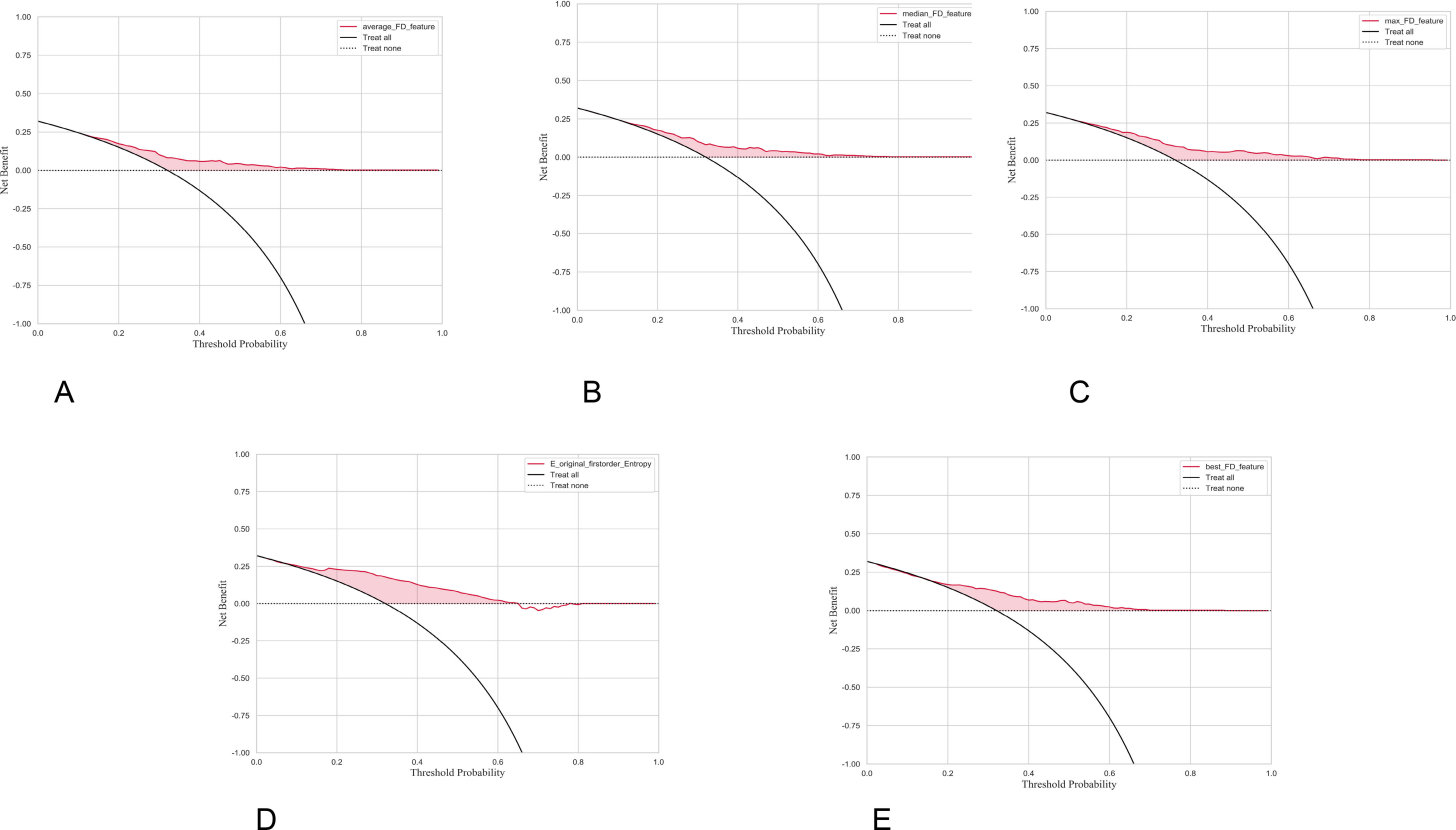
Five net benefit decision curve analysis line graphs labeled **(A–E)** compare different features’ clinical utility. Each graph includes three lines: the feature under analysis in pink, “Treat all” in solid black, and “Treat none” in dotted black. The yaxis shows net benefit from -1.0 to 1.0, and the x-axis shows threshold probability from 0.0 to 1.0. The pink area illustrates net benefit above treating none for various thresholds.Each panel analyzes a different feature, with **(A)** showing average_FD_feature, **(B)** median_FD_feature, **(C)** max_FD_feature, **(D)** E_original_firstorder_Entropy, and (E) best_FD_feature.

In terms of decision curve analysis (DCA), all single features yield positive net benefit compared to the “Treat none” strategy in the commonly used threshold probability range, and outperform the “Treat all” strategy in the low-to-medium threshold range, demonstrating their potential for clinical application (**Figure 5**). Among these, E_original_firstorder_Entropy has the widest coverage and the highest overall value of the net benefit curve, consistently outperforming the two baseline strategies at approximately P = 0.1-0.7. best_FD_feature has the second-highest net benefit, mainly concentrated in P = 0.1-0.6. The effective range of max_FD_feature extends slightly to P = 0.65, and is slightly better overall than average_FD_feature and median_FD_feature (both maintaining a stable net benefit at approximately P = 0.1-0.55). Comprehensive calibration and DCA results show that the entropy feature has advantages in both “probability estimation consistency” and “net clinical benefit,” with a wider range of applicable thresholds. The fractal dimension features provide complementary information of moderate strength and slightly higher threshold dependence, suggesting that they can be used as complementary features to enhance sensitivity and cover different decision threshold segments in joint modeling.

### 3.6 Subgroup analysis short-axis diameter

Regarding the detection capability for “micro-metastases/small volume lymph nodes (short-axis diameter below the commonly used 5 mm threshold)”, we divided the lymph nodes into two groups: short-axis <5 mm and short-axis ≥5 mm, and calculated the discriminative performance of the models within each group separately. The results showed that in the short-axis <5 mm subgroup, each model still maintained good discriminatory ability, with the model based on E_original_firstorder_Entropy performing the best, achieving an AUC of 0.813 and a sensitivity of 0.895 (**Table 4**), suggesting that this method has good detection ability for small lymph nodes below the 5 mm threshold (potential micro-metastasis related scenarios).

**Table 4:** Subgroup analysis of short-axis diameter.

Dataset	Model	Threshold	ACC	AUC	Sen sitivity	Specificity	NPV	PPV	F1
average_ FD_featu re_train	>5mm	0.295	0.614	0.714	0.759	0.546	0.827	0.441	0.558
median_F D_featur e_train	>5mm	0.311	0.636	0.711	0.699	0.606	0.810	0.455	0.551
max_FD_f eature_t rain	>5mm	0.288	0.628	0.729	0.789	0.553	0.847	0.454	0.576
E_origin al_first order_En tropy_tr ain	>5mm	0.266	0.766	0.812	0.894	0.705	0.934	0.589	0.710
best_FD_ feature_ train	>5mm	0.350	0.706	0.745	0.736	0.691	0.847	0.529	0.616

## 4 Discussion

In this study, we successfully developed an automatic segmentation model for lymph nodes adjacent to the right recurrent laryngeal nerve (RLN) using the nnU-Net framework. Additionally, we identified and validated five elasticity-related radiomic features by generating CT-based Differential Elasticity Maps (DEMs). By enabling non-invasive and reproducible analysis of tissue biomechanical properties, this method fills the gap in preoperative staging and is expected to optimize treatment strategies for patients with esophageal carcinoma (EC).

Accurate segmentation of the tumor region of interest (ROI) constitutes the foundation of tumor radiomics. Traditional approaches predominantly rely on manual ROI delineation, which is time-consuming, labor-intensive, and highly susceptible to subjective factors such as the operator’s experience and delineation habits (11). To address this limitation, we constructed an automated segmentation model based on the nnU-Net framework. As a fully automated deep learning architecture, nnU-Net has demonstrated remarkably high segmentation accuracy in medical image analysis tasks (16, 17). In our internal validation, the model achieved a Dice Similarity Coefficient (DSC) of 0.898 ± 0.024. A DSC value exceeding 0.8 is generally recognized as the threshold indicating high consistency between automatic segmentation results and the gold standard of manual segmentation (17). This outcome verifies the promising performance of our model in segmenting right RLN-adjacent lymph nodes, effectively overcoming the drawbacks of traditional manual segmentation, including poor reproducibility, cumbersome operation, and low efficiency. Nevertheless, the sole reliance on DSC may lead to insufficient evaluation of boundary discrepancies between segmented lymph nodes and the gold standard. Therefore, additional assessment metrics, such as the Hausdorff distance (18), should be incorporated in future evaluations to refine the accuracy of tissue boundary segmentation.

Radiomics enables non-invasive analysis of tumor heterogeneity by extracting multi-dimensional features (e.g., intensity, texture, shape) from medical images, including computed tomography (CT), ultrasound (US), and magnetic resonance imaging (MRI) (19, 20). Currently, radiomics has been widely applied in multiple domains, such as tumor phenotype prediction, therapeutic efficacy evaluation, and lymph node metastasis assessment (21, 22). Aerts et al. (23) and Wu et al. (24) have defined a variety of clinically significant radiomic features through systematic research, laying a solid foundation for the clinical translation of radiomic techniques. However, conventional radiomic features primarily capture tissue morphological and density characteristics, potentially overlooking alterations in tissue mechanical properties (e.g., elasticity changes) induced by pathological processes such as cancer cell metastasis (11). This insight has spurred the development of elastography techniques based on ultrasound and MRI (25–27). Building on this progress, our study innovatively employs CT-based Differential Elasticity Maps (CT-DEMs) to analyze lymph node metastasis by focusing on tissue elasticity features.

Initially, a total of 1652 features were extracted in this study, encompassing 91 elasticity features and 1561 contrast-enhanced CT radiomic features. To mitigate the risk of overfitting associated with the moderate sample size, we applied rigorous feature selection and exclusion criteria, eliminating features with P > 0.05 or an area under the receiver operating characteristic curve (AUC) ≤ 0.7. This filtering process yielded five key features: one original first-order entropy feature (E_original_firstorder_Entropy) and four fractal dimension indices (max_FD_feature, best_FD_feature, average_FD_feature, median_FD_feature). These selected features are essentially categorized as “mechanical texture” features, which do not describe the density or intensity of tumor tissue but rather the spatial heterogeneity of tissue hardness and elasticity distribution. Such mechanical features can more directly reflect alterations in the elastic properties of lymph node microstructure caused by pathological processes including cancer cell infiltration and stromal fibrosis (28), thereby providing complementary biological information that cannot be captured by traditional radiomic features.

Entropy serves as an indicator for measuring the randomness and complexity of image textures, capable of effectively reflecting tissue heterogeneity (23, 28). In DEMs, the entropy values of metastatic lymph nodes were significantly elevated, which is consistent with the findings of ultrasound elastography studies conducted by Wang et al. (29), indicating tissue elasticity disorder resulting from pathological processes such as necrosis or stromal hyperplasia. Fractal dimension is used to assess the multi-scale irregularity of tissues (30); its consistent elevation in the metastatic group suggests that lymph node architecture may be disrupted by tumor invasion. It should be noted that although these features exhibit superior discriminative power compared to traditional features, increased entropy values are not a specific indicator of metastatic lymph nodes in all cases—severe inflammatory or tuberculous lymph nodes may also present with elevated entropy. This potential confounding factor necessitates comprehensive judgment combined with other indicators in clinical practice. Furthermore, the decision curve analysis of this study demonstrated that within the clinically relevant threshold probability range of 10%–70%, the entropy feature-based assessment yielded higher clinical net benefits. This facilitates the identification of high-risk patients requiring neoadjuvant therapy or extensive lymph node dissection, while avoiding overtreatment of low-risk patients.

Compared with existing studies, the advantages and innovations of our research are mainly reflected in two aspects. First, addressing the limitations of Ou et al.’s study (30), which employed traditional CT radiomic features (e.g., shape, gray-level co-occurrence matrix textures) to predict EC lymph node metastasis but suffered from limited feature diversity and poor stability due to reliance on manual segmentation, our automated DEM-based approach directly correlates entropy and fractal dimension with tissue elasticity changes, thus resolving these issues. Second, in contrast to the nomogram prediction model constructed by Liu et al. (31), our method enables non-invasive preoperative assessment without the need for intraoperative pathological data and exhibits superior performance in identifying micrometastases with a short-axis diameter < 5 mm. However, it should be objectively noted that the AUC value of our model (0.814) still has room for improvement compared with that of advanced multi-modal radiomic models (AUC > 0.85) (32, 33). This discrepancy may be attributed to the inclusion of only CT single-modal data and the lack of integration with clinical indicators (e.g., tumor differentiation grade, smoking history).Therefore, readers should interpret our research results with caution. Future studies should encourage the combination of digital elevation model (DEM) features with multimodal data, such as tumor habitat typing in images (e.g., necrotic, actively proliferating, hypoxic sub-regions) to capture the impact of heterogeneity on entropy and fractal features. On the other hand, spatial transcriptome sequencing should be conducted in newly collected biopsy samples to analyze the gene expression profiles in different habitat regions (immune infiltration, metabolic pathways, etc.), thereby linking imaging phenotypes with molecular mechanisms and screening for explainable markers; to achieve higher diagnostic accuracy.

Despite its merits, this study has several limitations. First, as a single-center retrospective study, all enrolled cases were sourced from a single medical institution. Homogeneity in variables such as patient geographical characteristics and institutional diagnostic protocols may introduce selection bias, potentially limiting the applicability of the model in diverse clinical settings. Second, the focus on single-feature analysis facilitates the clarification of the independent diagnostic value of each elasticity feature, but it may underestimate the synergistic effects with other clinical variables (e.g., tumor diameter). Third, no subgroup analysis was performed to evaluate the impact of different CT scanners and scanning parameters; variations in scanning protocols across different manufacturers may affect the stability of DEM features (34), which is a critical issue to be addressed for clinical translation. Finally, the study did not include samples of benign lymph node abnormalities caused by inflammation, tuberculosis, or other benign conditions, precluding verification of the model’s efficacy in differentiating benign from malignant lymph nodes.

The translation of this research into clinical practice holds considerable feasibility. Given the open-source nature of the nnU-Net framework and the CT compatibility of DEM technology, this method is expected to be integrated into clinical medical systems to enable real-time analysis. Therefore, future research should prioritize the implementation of multi-center prospective studies, enrolling cases from different regions and using various CT scanners to further validate the generalizability of the model. Second, efforts should be made to construct a comprehensive prediction model integrating DEM elasticity features, multiple clinical parameters, and multi-modal imaging data (CT + MRI) to enhance diagnostic performance. Finally, the sample spectrum should be expanded to include benign lymph node lesions, thereby improving the model’s differential diagnostic capability.

Nevertheless, we acknowledge that LASSO and other regularization techniques provide valuable tools for high−dimensional feature selection and model penalization. In future investigations, integrating DEM−derived features with LASSO−based models may further improve predictive performance, especially as larger multi−center cohorts become available. Such an approach could help balance model complexity and generalizability while preserving the biomechanical interpretability of the DEM features.

## 5 Conclusion

In conclusion, by integrating nnU-Net-based automatic segmentation and CT-DEM technology, this study achieves non-invasive and accurate prediction of right RLN-adjacent lymph node metastasis in EC. The proposed mechanical texture features provide a valuable complement to traditional radiomics. Despite certain limitations, this study offers novel insights and methodologies for precise preoperative staging and personalized treatment planning in EC patients.

## Data Availability

The original contributions presented in the study are included in the article/supplementary material. Further inquiries can be directed to the corresponding author.

## References

[1] Bray F Laversanne M Sung H Ferlay J Siegel RL Soerjomataram I . Global cancer statistics 2022: GLOBOCAN estimates of incidence and mortality worldwide for 36 cancers in 185 countries. CA Cancer J Clin. (2024) 74:229–63. doi: 10.3322/caac.21834. PMID: 38572751

[2] Zhu H Ma X Ye T Wang H Wang Z Liu Q . Esophageal cancer in China: Practice and research in the new era. Intl J Cancer. (2023) 152:1741–51. doi: 10.1002/ijc.34301. PMID: 36151861

[3] Wang Y Zhu L Xia W Wang F . Anatomy of lymphatic drainage of the esophagus and lymph node metastasis of thoracic esophageal cancer. CMAR. (2018) Volume 10:6295–303. doi: 10.2147/CMAR.S182436. PMID: 30568491 PMC6267772

[4] Altorki NK Zhou XK Stiles B Port JL Paul S Lee PC . Total number of resected lymph nodes predicts survival in esophageal cancer. Ann Surg. (2008) 248:221–6. doi: 10.1097/SLA.0b013e31817bbe59. PMID: 18650631

[5] Greenstein AJ Litle VR Swanson SJ Divino CM Packer S Wisnivesky JP . Prognostic significance of the number of lymph node metastases in esophageal cancer. J Am Coll Surgeons. (2008) 206:239–46. doi: 10.1016/j.jamcollsurg.2007.09.003. PMID: 18222375

[6] Shen C Liu Z Wang Z Guo J Zhang H Wang Y . Building CT radiomics based nomogram for preoperative esophageal cancer patients lymph node metastasis prediction. Transl Oncol. (2018) 11:815–24. doi: 10.1016/j.tranon.2018.04.005. PMID: 29727831 PMC6154864

[7] Cho H-H Kim CK Park H . Overview of radiomics in prostate imaging and future directions. Br J Radiol. (2022) 95:20210539. doi: 10.1259/bjr.20210539. PMID: 34797688 PMC8978251

[8] Lambin P Rios-Velazquez E Leijenaar R Carvalho S van Stiphout RG Granton P . Radiomics: Extracting more information from medical images using advanced feature analysis. Eur J Cancer. (2012) 48:441–6. doi: 10.1016/j.ejca.2011.11.036. PMID: 22257792 PMC4533986

[9] Li X Liu Q Hu B Xu J Huang C Liu F . A computed tomography-based clinical-radiomics model for prediction of lymph node metastasis in esophageal carcinoma. J Cancer Res Ther. (2021) 17:1665–71. doi: 10.4103/jcrt.jcrt_1755_21. PMID: 35381737

[10] Jannatdoust P Valizadeh P Pahlevan-Fallahy MT Hassankhani A Amoukhteh M Behrouzieh S . Diagnostic accuracy of CT-based radiomics and deep learning for predicting lymph node metastasis in esophageal cancer. Clin Imaging. (2024) 113:110225. doi: 10.1016/j.clinimag.2024.110225. PMID: 38905878

[11] Pham DL Xu C Prince JL . Current methods in medical image segmentation. Annu Rev BioMed Eng. (2000) 2:315–37. doi: 10.1146/annurev.bioeng.2.1.315. PMID: 11701515

[12] Cao W Pomeroy MJ Liang Z Gao Y Shi Y Tan J . Lesion classification by model-based feature extraction: A differential affine invariant model of soft tissue elasticity in CT images. J Digit Imaging Inform Med. (2024) 38:804–18. doi: 10.1007/s10278-024-01178-8. PMID: 39164453 PMC11950485

[13] Weng WC Huang CW Su CC Mukundan A Karmakar R Chen TH . Optimizing esophageal cancer diagnosis with computer-aided detection by YOLO models combined with hyperspectral imaging. Diagnostics. (2025) 15:1686. doi: 10.3390/diagnostics15131686. PMID: 40647685 PMC12248576

[14] Chang L-J . Evaluation of spectral imaging for early esophageal cancer detection. Cancers. (2025) 17:2049. doi: 10.3390/cancers17122049. PMID: 40563697 PMC12190529

[15] Chen Y-C Karmakar R Mukundan A Huang C-W Weng W-C Wang H-C . Evaluation of band selection for spectrum-aided visual enhancer (SAVE) for esophageal cancer detection. J Cancer. (2025) 16:470–8. doi: 10.7150/jca.102759. PMID: 39744490 PMC11685682

[16] Vossough A . Training and comparison of nnU-Net and DeepMedic methods for autosegmentation of pediatric brain tumors. AJNR Am J Neuroradiol. (2024) 45:1081–9. doi: 10.3174/ajnr.A8293. PMID: 38724204 PMC11383404

[17] Ferrante M Rinaldi L Botta F Hu X Dolp A Minotti M . Application of nnU-Net for automatic segmentation of lung lesions on CT images and its implication for radiomic models. JCM. (2022) 11:7334. doi: 10.3390/jcm11247334. PMID: 36555950 PMC9784875

[18] Pogson EM Begg J Jameson MG Dempsey C Latty D Batumalai V . A phantom assessment of achievable contouring concordance across multiple treatment planning systems. Radiother Oncol. (2015) 117:438–41. doi: 10.1016/j.radonc.2015.09.022. PMID: 26427804

[19] Scapicchio C Gabelloni M Barucci A Cioni D Saba L Neri E . A deep look into radiomics. Radiol Med. (2021) 126:1296–311. doi: 10.1007/s11547-021-01389-x. PMID: 34213702 PMC8520512

[20] Liu Z Wang S Dong D Wei J Fang C Zhou X . The applications of radiomics in precision diagnosis and treatment of oncology: Opportunities and challenges. Theranostics. (2019) 9:1303–22. doi: 10.7150/thno.30309. PMID: 30867832 PMC6401507

[21] Coroller TP Agrawal V Narayan V Hou Y Grossmann P Lee SW . Radiomic phenotype features predict pathological response in non-small cell lung cancer. Radiother Oncol. (2016) 119:480–6. doi: 10.1016/j.radonc.2016.04.004. PMID: 27085484 PMC4930885

[22] Li Y Deng J Ma X Li W Wang Z . Diagnostic accuracy of CT and PET/CT radiomics in predicting lymph node metastasis in non-small cell lung cancer. Eur Radiol. (2024) 35:1966–79. doi: 10.1007/s00330-024-11036-4. PMID: 39223336

[23] Aerts HJ Velazquez ER Leijenaar RT Parmar C Grossmann P Carvalho S . Decoding tumour phenotype by noninvasive imaging using a quantitative radiomics approach. Nat Commun. (2014) 5:4006. doi: 10.1038/ncomms5006. PMID: 24892406 PMC4059926

[24] Wu YP Wu L Ou J Cao JM Fu MY Chen TW . Preoperative CT radiomics of esophageal squamous cell carcinoma and lymph node to predict nodal disease with a high diagnostic capability. Eur J Radiol. (2024) 170:111197. doi: 10.1016/j.ejrad.2023.111197. PMID: 37992611

[25] Guo J Savic LJ Hillebrandt KH Sack I . MR elastography in cancer. Invest Radiol. (2023) 58:578–86. doi: 10.1097/RLI.0000000000000971. PMID: 36897804

[26] Good DW Stewart GD Hammer S Scanlan P Shu W Phipps S . Elasticity as a biomarker for prostate cancer: a systematic review. BJU Int. (2014) 113:523–34. doi: 10.1111/bju.12236. PMID: 23905869

[27] Knabe M Günter E Ell C Pech O . Can EUS elastography improve lymph node staging in esophageal cancer? Surg Endosc. (2013) 27:1196–202. doi: 10.1007/s00464-012-2575-y. PMID: 23093233

[28] Radman BA Alhameed AMM Shu G Yin G Wang M . Cellular elasticity in cancer: a review of altered biomechanical features. J Mater Chem B. (2024) 12:5299–324. doi: 10.1039/D4TB00328D. PMID: 38742281

[29] Wang B Guo Q Wang JY Yu Y Yi AJ Cui XW . Ultrasound elastography for the evaluation of lymph nodes. Front Oncol. (2021) 11:714660. doi: 10.3389/fonc.2021.714660. PMID: 34485150 PMC8415874

[30] Foroutan-pour K Dutilleul P Smith DL . Advances in the implementation of the box-counting method of fractal dimension estimation. Appl Math Comput. (1999) 105:195–210. doi: 10.1016/S0096-3003(98)10096-6

[31] Liu Y Zou Z-Q Xiao J Zhang M Yuan L Zhao X-G . A nomogram prediction model for recurrent laryngeal nerve lymph node metastasis in thoracic oesophageal squamous cell carcinoma. J Thorac Dis. (2019) 11:2868–77. doi: 10.21037/jtd.2019.06.46. PMID: 31463116 PMC6688001

[32] Warkentin MT Al-Sawaihey H Lam S Liu G Diergaarde B Yuan JM . Radiomics analysis to predict pulmonary nodule Malignancy using machine learning approaches. Thorax. (2024) 79:307–15. doi: 10.1136/thorax-2023-220226. PMID: 38195644 PMC10947877

[33] Wang X Song J Qiu Q Su Y Wang L Cao X . A stacked multimodality model based on functional MRI features and deep learning radiomics for predicting the early response to radiotherapy in nasopharyngeal carcinoma. Acad Radiol. (2025) 32:1631–44. doi: 10.1016/j.acra.2024.10.011. PMID: 39496536

[34] Levi R Mollura M Savini G Garoli F Battaglia M Ammirabile A . A reference framework for standardization and harmonization of CT radiomics features on cadaveric sample. Sci Rep. (2024) 14:19259. doi: 10.1038/s41598-024-68158-4. PMID: 39164314 PMC11336160

